# Supplementary material for: Degradation of Tetracycline Hydrochloride by Cu-Doped MIL-101(Fe) Loaded Diatomite Heterogeneous Fenton Catalyst
Source: Nanomaterials (Basel). 2022 Feb 28;12(5):811. doi: 10.3390/nano12050811 (PMC8912278; doi:10.3390/nano12050811)
Supplement: Supplementary file 1 [file nanomaterials-12-00811-s001.zip › nanomaterials-1565189 supplementary.pdf]

nanomaterials

Supporting information

Degradation of Tetracycline Hydrochloride by Cu-Doped MIL-101(Fe) Loaded Diatomite Heterogeneous Fenton Catalyst

Kang-Ping Cui [1,*], Yu-Ying He [1], Kai-Jie Xu [1], Yu Zhang [2], Chang-Bin Chen [3], Zheng-Jiang Xu [3] and Xing Chen [1,2,*]

[1] School of Resource and Environmental Engineering, Hefei University of Technology, Hefei 230009, China; heyuying131@163.com; (Y.-Y.H.); XUkaijie@mail.hfut.edu.cn (K.-J.X.)
[2] Key Lab of Aerospace Structural Parts Forming Technology and Equipment of Anhui Province, Institute of Industry and Equipment Technology, Hefei University of Technology, Hefei 230009, China; zhang-yu70714@163.com
[3] Anqing Shuguang Chemical Co., Ltd., Anqing 246003, China; chenchangbin19@163.com (C.-B.C.); xuzhengjiang19@163.com (Z.-J.X.)
* Correspondence: cuikangping@hfut.edu.cn (K.-P.C.); xingchen@hfut.edu.cn (X.C.)

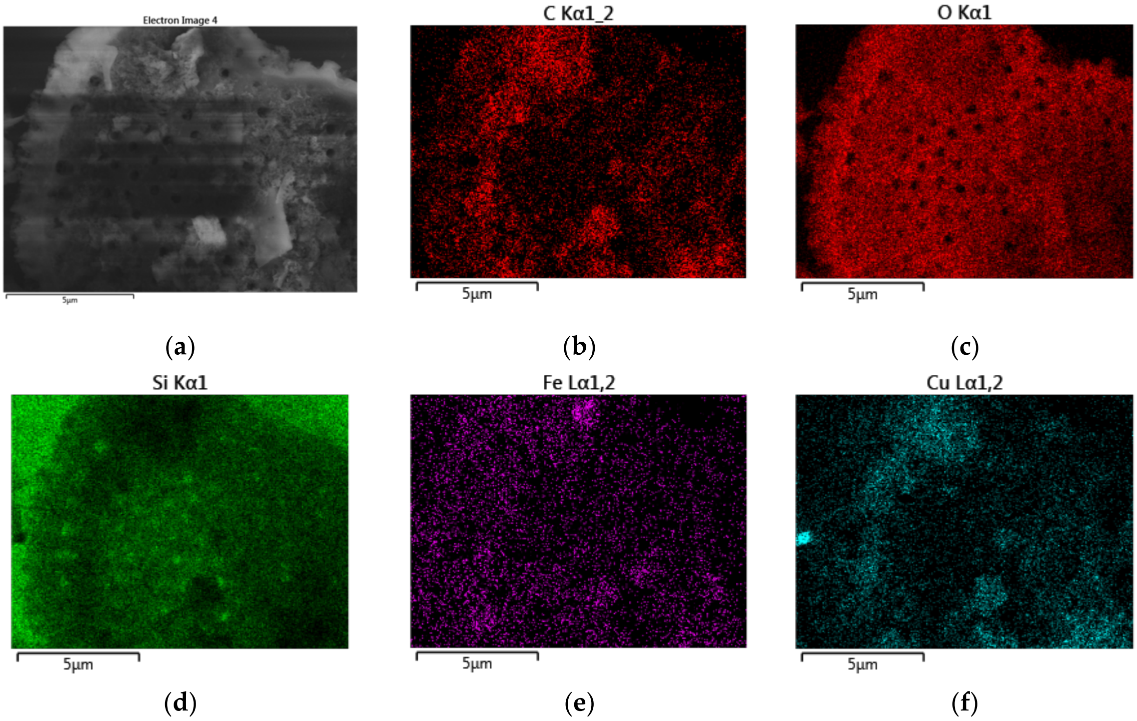

Figure S1. (**a**)EDXS elemental mapping for $Fe_{0.25}Cu_{0.75}$(BDC)@DE confirmed the presence of (**b**) C, (**c**) O (**d**) Si (**e**) Fe and (**f**) Cu.

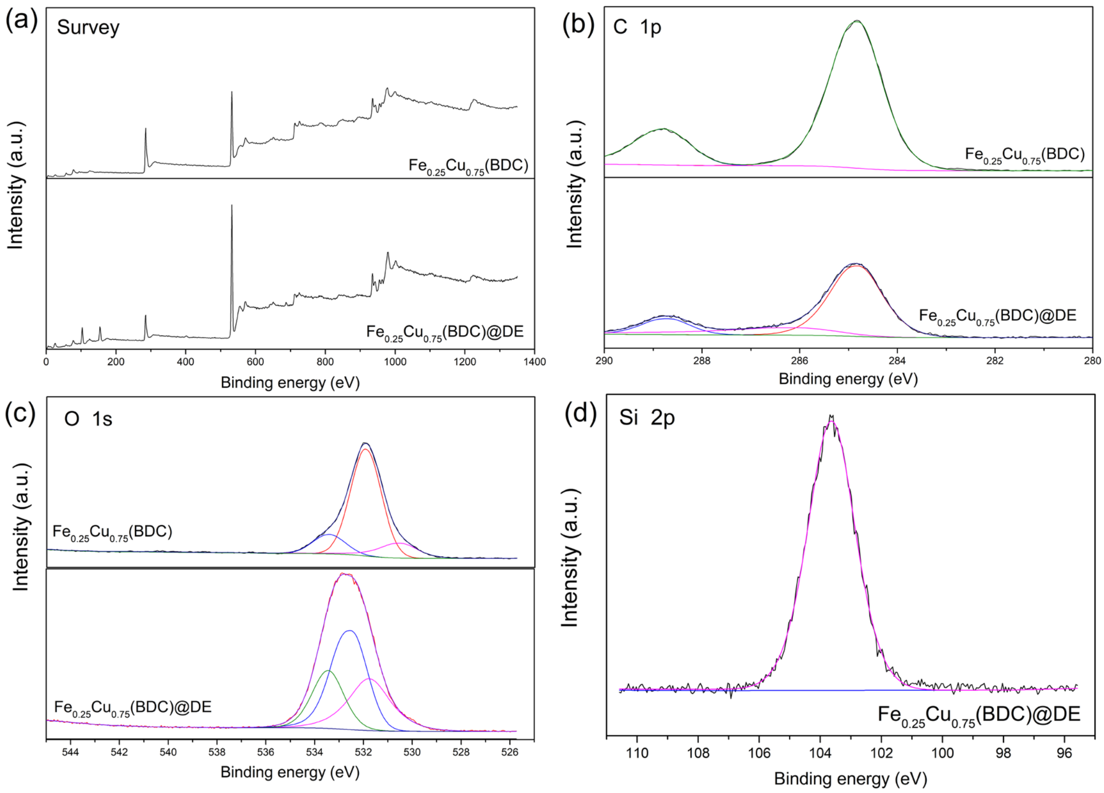

Figure S2. (**a**) XPS survey spectrum and high-resolution XPS spectra of (**b**) C 1p, (**c**) O 1s for the $Fe_{0.25}Cu_{0.75}$(BDC) and $Fe_{0.25}Cu_{0.75}$(BDC)@DE nanoparticles, and (**d**) Si 2p. for $Fe_{0.25}Cu_{0.75}$(BDC)@DE nanoparticles.

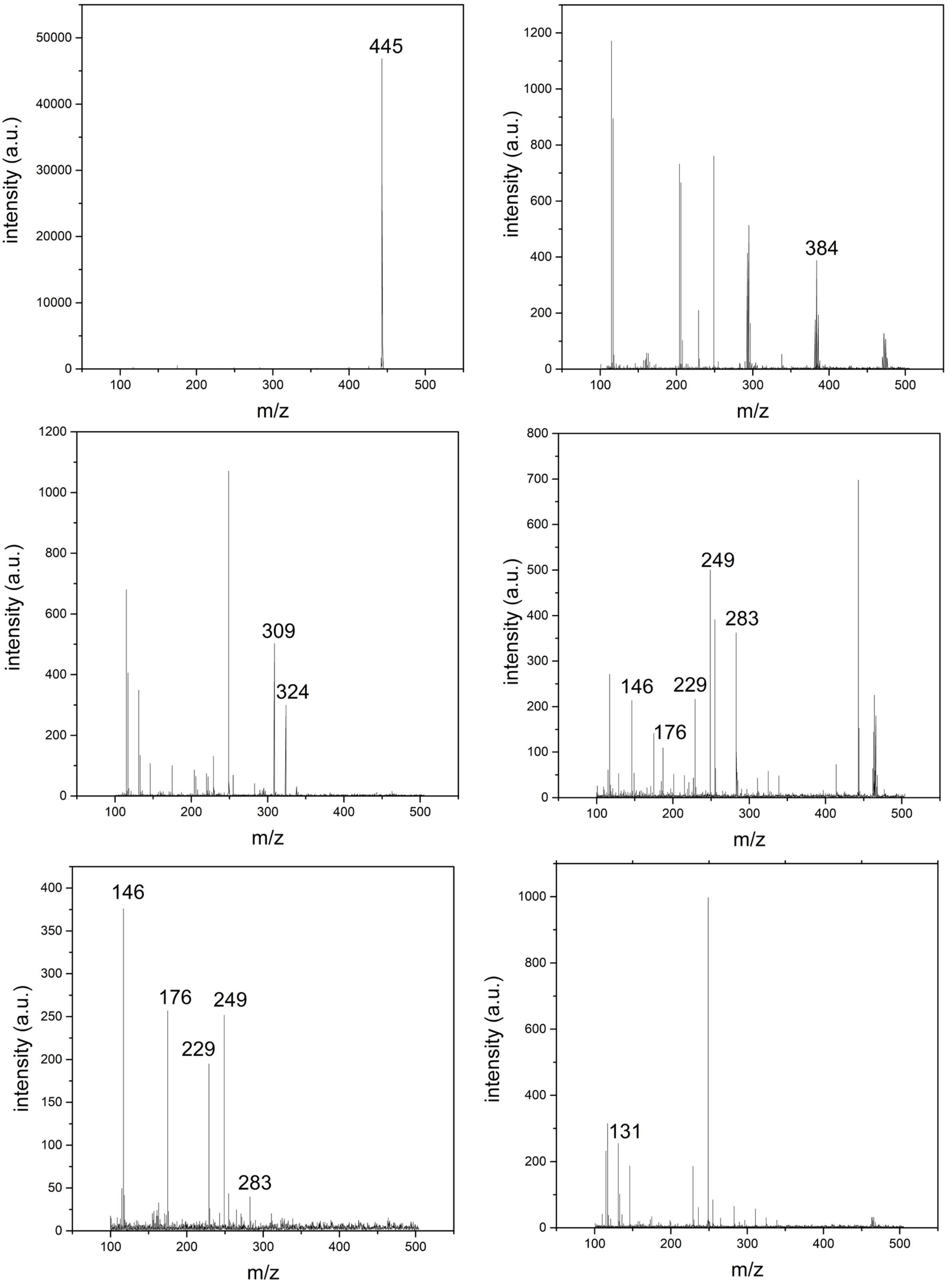

Figure S3. Mass spectrum of TC and its degradation intermediates.
